# Unilateral Eccentric Contraction of the Plantarflexors Leads to Bilateral Alterations in Leg Dexterity

**DOI:** 10.3389/fphys.2016.00582

**Published:** 2016-11-30

**Authors:** Akira Nagamori, Francisco J. Valero-Cuevas, James M. Finley

**Affiliations:** ^1^Division of Biokinesiology and Physical Therapy, University of Southern CaliforniaLos Angeles, CA, USA; ^2^Department of Biomedical Engineering, University of Southern CaliforniaLos Angeles, CA, USA

**Keywords:** sensorimotor function, dexterity, lower extremity control, eccentric contraction, muscle proprioception, spinal feedback control

## Abstract

Eccentric contractions can affect musculotendon mechanical properties and disrupt muscle proprioception, but their behavioral consequences are poorly understood. We tested whether repeated eccentric contractions of plantarflexor muscles of one leg affected the dexterity of either leg. Twenty healthy male subjects (27.3 ± 4.0 yrs) compressed a compliant and slender spring prone to buckling with each isolated leg. The maximal instability they could control (i.e., the maximal average sustained compression force, or lower extremity dexterity force, LED_force_) quantified the dexterity of each leg. We found that eccentric contractions did not affect LED_force_, but reduced force variability (LED_SD_). Surprisingly, LED_force_ increased in the non-exposed, contralateral leg. These effects were specific to exposure to eccentric contractions because an effort-matched exposure to walking did not affect leg dexterity. In the exposed leg, eccentric contractions (i) reduced voluntary error corrections during spring compressions (i.e., reduced 0.5–4 Hz power of LED_force_); (ii) did not change spinal excitability (i.e., unaffected H-reflexes); and (iii) changed the structure of the neural drive to the α-motoneuron pool (i.e., reduced EMG power within the 4–8 Hz physiological tremor band). These results suggest that repeated eccentric contractions alter the feedback control for dexterity in the exposed leg by reducing muscle spindle sensitivity. Moreover, the unexpected improvement in LED_force_ in the non-exposed contralateral leg was likely a consequence of crossed-effects on its spinal and supraspinal feedback control. We discuss the implications of these bilateral effects of unilateral eccentric contractions, their effect on spinal and supraspinal control of dynamic foot-ground interactions, and their potential to facilitate rehabilitation from musculoskeletal and neuromotor impairments.

## 1. Introduction

Successful performance of balance and locomotor tasks is mediated by the ability of the leg to control force vectors to regulate dynamic foot-ground interactions, which has been referred to as dexterity (Valero-Cuevas et al., 2003; Lyle et al., 2013; Lawrence et al., 2014). We define dexterity as per the Strength-Dexterity paradigm (Valero-Cuevas et al., 2003), where a limb attempts to fully compress a compliant and slender spring prone to buckling. The ability to prevent buckling by controlling the ensuing instability is the dexterity score, which quantifies the integrity of the sensorimotor system for dynamical control of the limb. We have found that dexterity can be considered a functional domain distinct from strength and multijoint coordination (Lawrence et al., 2015a,b). The dexterity test for the leg has also been associated with athletic skills such as agility (Lyle et al., 2015). Such dynamic regulation of foot-ground interactions becomes an especially critical skill during highly dynamic tasks such as single leg landing (Brown et al., 2004; Ross and Guskiewicz, 2004). Accordingly, our previous work demonstrated reduced leg dexterity in female athletes which may contribute to their altered landing biomechanics and expose them to greater risks of non-contact anterior cruciate ligament (ACL) injuries (Lawrence et al., 2014; Lyle et al., 2014; Lawrence et al., 2016). Impairments in leg dexterity may also lead to reductions in mobility in individuals with hemiparesis (Higginson et al., 2006) and higher risks of other injuries such as repeated ankle sprains (Arnold et al., 2009; Kobayashi and Gamada, 2014). Therefore, improving our understanding of the sensorimotor mechanisms that enable dynamic foot-ground interactions may be important for elucidating mechanisms of impaired balance and locomotion in health and disease.

There are multiple long- and short-latency sensorimotor mechanisms thought to contribute to the ability to dynamically control foot-ground interactions and maintain balance during locomotion. These include vestibular function and visual acuity, strength, muscle coordination, and somatosensory system including proprioception (Dietz et al., 1980; Mauritz and Dietz, 1980; Woollacott et al., 1985; Manchester et al., 1989; Horak et al., 1990; Hay et al., 1996; Kavounoudias et al., 1999; Hassan et al., 2001; Speers et al., 2002; Marigold et al., 2004). Among these, muscle proprioception likely plays a significant role in the feedback control of dexterity. For example, previous studies have shown that short- and medium-latency reflex responses are modulated according to nature of the dynamic interface between the foot and environment (i.e., rigid vs. compliant vs. unstable) (Finley et al., 2012, 2013). These results suggest that tuning of sensorimotor function through modulation of proprioception may be critical for controlling dexterity in different environments. Elucidating the role of proprioception in the control of lower extremity dexterity requires experimental paradigms capable of manipulating proprioception. While this can be accomplished using techniques such as ischemic blocking (Dietz et al., 1980; Mauritz and Dietz, 1980) or prolonged tendon vibration (Hay et al., 1996; Kavounoudias et al., 1999), we elected to use non-fatiguing eccentric contractions because such contraction are thought to be a natural means to manipulate muscle proprioception—and because they are relevant to activities of daily living and athletic performance.

Eccentric contractions are known to have unique physiological effect on muscle proprioception compared to other contraction types and movements. First, eccentric contraction provides more afferent input through muscle spindles compared to isometric or concentric contractions, as muscle stretch associated with the movement leads to greater spindle activity (Burke et al., 1978). Such augmentation of proprioceptive input is known to have profound effects on both spinal and supraspinal processing of afferent feedback (Siggelkow et al., 1999; Rosenkranz and Rothwell, 2004, 2006; Marconi et al., 2008) which may impact the control of dynamic foot-ground interactions. Moreover, the augmented proprioceptive input during eccentric contractions may lead to bilateral alterations in neuromuscular control, as shown in acute and chronic strength improvements in the contralateral leg (Grabiner and Owings, 1999; Hortobágyi et al., 2003; Howatson et al., 2011).

Therefore, we tested the hypothesis that manipulation of muscle proprioception by unilateral exposure to an acute bout of eccentric contractions affects the control of dynamic foot-ground interactions (i.e., leg dexterity). Our results using leg dexterity avoid confounds such as strength, vestibular function, visual acuity, bilateral limb coordination, and upper-and-lower body coordination (Lawrence et al., 2014; Lyle et al., 2014, 2015). The changes in leg dexterity we find in response to eccentric contractions provide new insights into how muscle proprioception is integrated into the feedback control of dexterity and how unilateral eccentric contractions can lead to bilateral improvements in sensorimotor capacity.

## 2. Methods

### 2.1. Subjects

Twenty healthy male subjects (age = 27.3 ± 4.0 years, height = 1.77 ± 0.08 m, body weight = 75.9 ± 13.9 kg) consented to participate in this study. Only male subjects were recruited in this study in order to account for potential confounding effects of sex on leg dexterity (Lawrence et al., 2014; Lyle et al., 2014). This study was approved by the Health Science Campus Institutional Review Board at the University of Southern California and all study procedures conformed to the guidelines outlined in the Declaration of Helsinki.

### 2.2. Lower extremity dexterity test protocol

**Figure 1:**
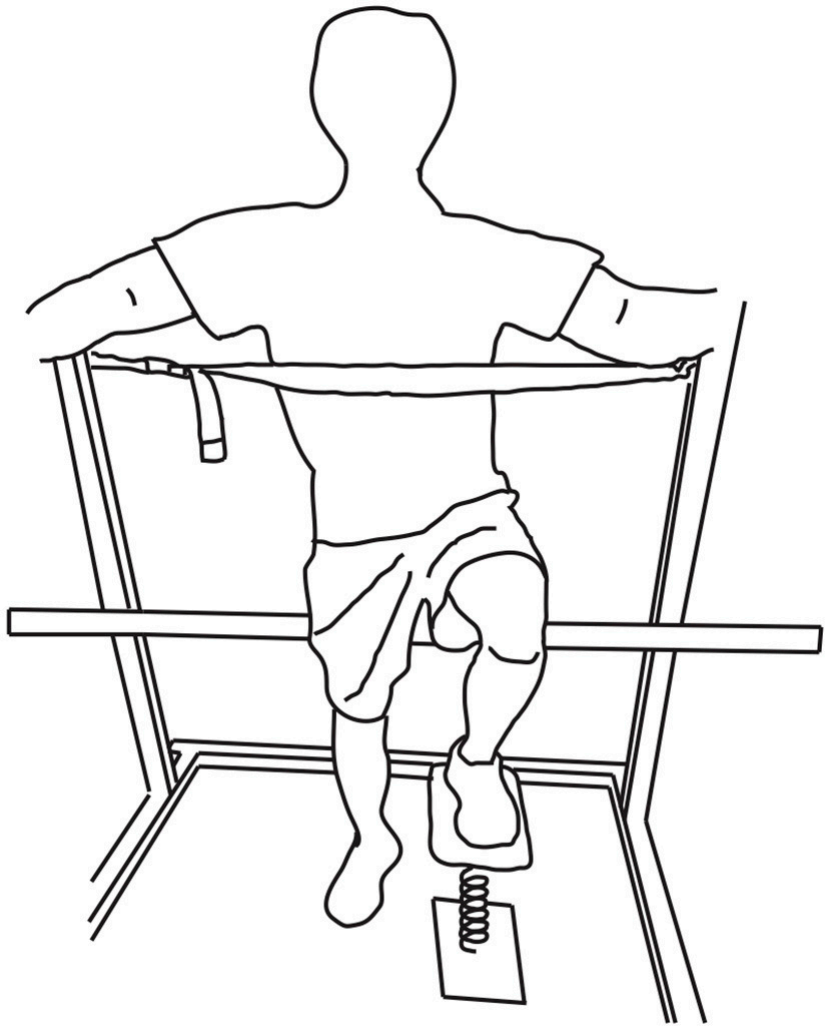
**LED test positioning**. Subjects placed the tested leg on the LED test system while partly seated on a bicycle seat. The height of the bicycle seat was adjusted for the contralateral foot to make complete contact with the ground. A strap at the height of the xiphoid process and safety bars were used for additional support.

This study was designed to test effects of low-intensity, repetitive eccentric contractions on the ability to regulate dynamic foot-ground interactions (i.e., leg dexterity). This ability was assessed using the Strength-Dexterity test adapted for the isolated leg (Neuromuscular Dynamics, LLC, La Crescenta, CA). Detailed description of the lower extremity dexterity (LED) test can be found elsewhere (Lyle et al., 2013; Lawrence et al., 2014). In short, this test evaluates participant's ability to compress a compliant and slender spring that is prone to buckling at low force magnitudes. For this test, a helical compression spring was mounted on a uniaxial force transducer as shown in Figure 1. The spring becomes increasingly unstable as it is being compressed, and the maximal sustained compressions at the edge of instability for each subject quantifies the maximal level of foot-ground instability that the isolated leg can control while producing low forces. Importantly, the spring is designed such that maximal sustained compression requires c. 20% of body weight. Full compression of the spring is impossible due to the ever-increasing instability.

Subjects were instructed to compress the spring as far as possible and hold that maximal level of compression for at least 5 s. As in prior work, subjects were allowed familiarization trials as needed (usually 5 trials), and then performed at least 10 trials for each leg. Subjects were partly seated on a bicycle saddle while equally distributing their body weight between the contralateral leg and the bicycle saddle (Figure 1). This is a standardized posture consistent with previous studies (Lyle et al., 2013; Lawrence et al., 2014). In order to minimize the use of the contralateral leg and trunk during this isolated leg test, subjects were asked to lean on a strap at the height of the xiphoid process and to hold safety bars on a squat rack for additional support.

### 2.3. Data acquisition and processing

#### 2.3.1. Maximal sustained spring compression

As in prior work, the compression force was sampled at 2000 Hz through USB-DAQ (Measurement Computing, Norton, MA) and recorded using a custom-built program in MATLAB (The Mathworks, Natick, MA). Raw LED compression force data were visually inspected and the hold phases were manually selected. Individual hold phases were truncated to have equal duration (c. 3 s) across trials to facilitate analysis. Five trials where subjects achieved their highest mean LED compression force during the hold phase (LED_force_) were used for further analysis. Also, standard deviation of LED_force_ during the hold phase (LED_SD_) from the same trials were computed to quantify the degree of force variability. The mean of five trails for LED_force_ and LED_SD_ was used as a measure of subjects' performance.

The dynamics of force variability during the hold phases was also quantified using frequency domain analysis as in Ko et al. (2015). The mean force was removed from raw compression force data. Power spectral density was estimated for each trial using 2-s Gaussian moving window with 50% overlap with frequency resolution of 0.1 Hz. The average power spectrum of the best five trials was computed for each condition. It was then divided into two distinct frequency bands (0.5–4 and 4–8 Hz), and the mean power within each band was calculated (LED_0.5−4Hz_ and LED_4−8Hz_, respectively). The power contained in the 0.5–4 Hz band is considered to reflect movement associated with voluntary error corrections (Squeri et al., 2010). On the other hand, the 4–8 Hz band, often referred to physiological tremor, has been suggested to contain the information associated with involuntary movement such as reflexive movement (Iaizzo and Pozos, 1982; Pozos et al., 1982).

#### 2.3.2. Electromyography

We recorded the electromyographic (EMG) activity of the *soleus* and *tibialis anterior* (TA) muscles using self-adhesive surface bipolar electrodes (inter-electrodes of 22 mm, Myotronics Inc., Kent, WA). The electrode placement for each muscle was as follows. Soleus: 1–2 cm below the inferior aspect of the medial head of the *gastrocnemius*, and TA: 1 cm lateral to the tibia at one third of the line connecting the fibula head and lateral malleolus (Campanini et al., 2007). The skin areas for electrode placement were cleaned by abrasive jell and alcohol. EMG signals were sampled at 2000 Hz, band-pass filtered between 50 and 1000 Hz using a 4th order Butterworth filter and notch-filtered at 60 Hz. The overall magnitude of EMG activity was quantified by the root-mean square amplitude (EMG_RMS_).

Frequency domain analysis was also performed on the EMG signals of the soleus and TA to better understand neural mechanisms responsible for changes in LED test performance. We first rectified the band-pass filtered EMG signals and normalized the rectified signals to their respective unit variance. We then calculated power in the 0.5–4 Hz and 4–8 Hz frequency bands (EMG_0.5−4Hz_, EMG_4−8Hz_, respectively) for the same time periods of sustained spring compression.

The level of coactivation between the soleus and TA during the periods of sustained spring compressions for the LED test was also computed to examine potential changes in a feedforward strategy through cocontraction (Finley et al., 2012). The band-pass filtered and rectified EMG signals were further low-pass filtered with a 4th order Butterworth filter at 50 Hz before calculating cocontraction. Cocontraction was calculated using the equation below (Schmitt and Rudolph, 2008),

(1) $$\frac{\sum_{i=1}^{n}\left(\frac{EMGlow_{i}}{EMGhigh_{i}}\right)\times (EMGlow_{i}+EMGhigh_{i})}{n}$$

where *i* is time step, *n* is the number of total samples in a trial (i.e., sustained compression), and *EMGlow*_*i*_ and *EMGhigh*_*i*_ are the lower and higher of the *EMG* amplitudes of the soleus and TA at that time step, respectively.

#### 2.3.3. Hoffmann's reflex

**Figure 2:**
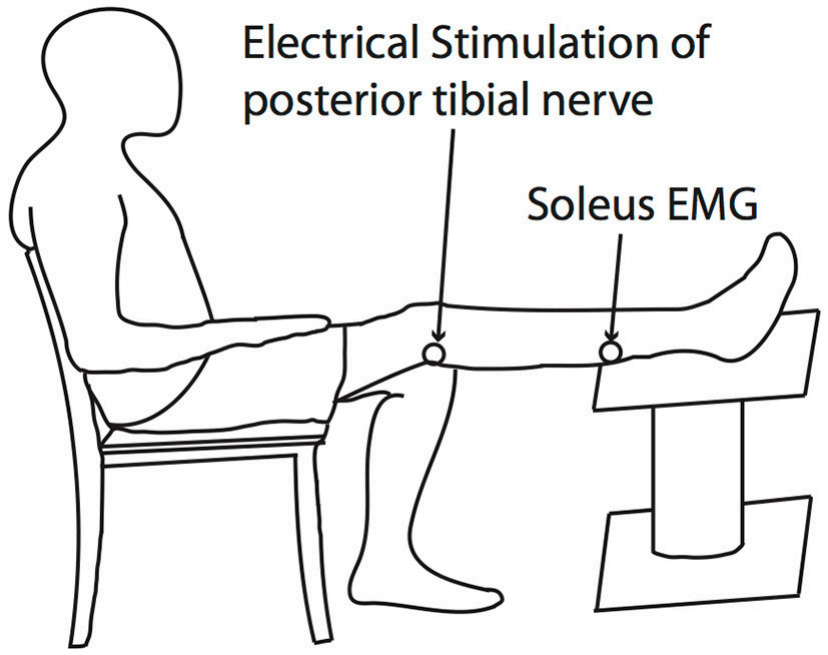
**H-reflex testing posture**. The H-reflex of the soleus was obtained by applying 1ms square electrical pulses to the posterior tibial nerve through a bipolar stimulation electrode using a constant current stimulator.

The Hoffmann reflex (H-reflex) is a measure of the gain of Ia afferent signal onto alpha-motoneurons modulated at the level of spinal cord. We used this outcome measure to interrogate possible effects of the experimental exposures on the gain of spinal reflex pathways. The H-reflex of the soleus was obtained by applying 1 ms square electrical pulses to the posterior tibial nerve through a bipolar stimulation electrode using a constant current stimulator (Digitimer Ltd., Hertfordshire, England). H-reflexes and motor responses (M-waves) were identified from raw EMG signals, and the peak-to-peak amplitudes of those responses were calculated within time windows for each response, which were manually-selected using an interactive function in MATLAB. During H-reflex measurement, subjects were seated with their tested leg completely extended. They were instructed to remain completely relaxed during the course of measurements (Figure 2).

Recruitment curves for H-reflex and M-wave were obtained by calculating their respective peak-to-peak amplitudes at increasing stimulus intensities up to the intensity that induced the maximal M-wave (M_max_). M_max_ was identified at the point when an increase in stimulus intensity no longer produced a further increase in M-wave amplitude. Test H-reflexes were then measured in the presence of a small M-wave, whose peak-to-peak amplitude corresponded to 10% M_max_. At least 10 H-reflex responses (H) were obtained and averaged for each condition. Averaged H-reflex amplitudes were normalized to each subject's M_max_ (i.e., H/M_max_) and used for statistical analysis described below.

#### 2.3.4. Eccentric contractions and plantarflexion maximal voluntary isometric contractions

**Figure 3:**
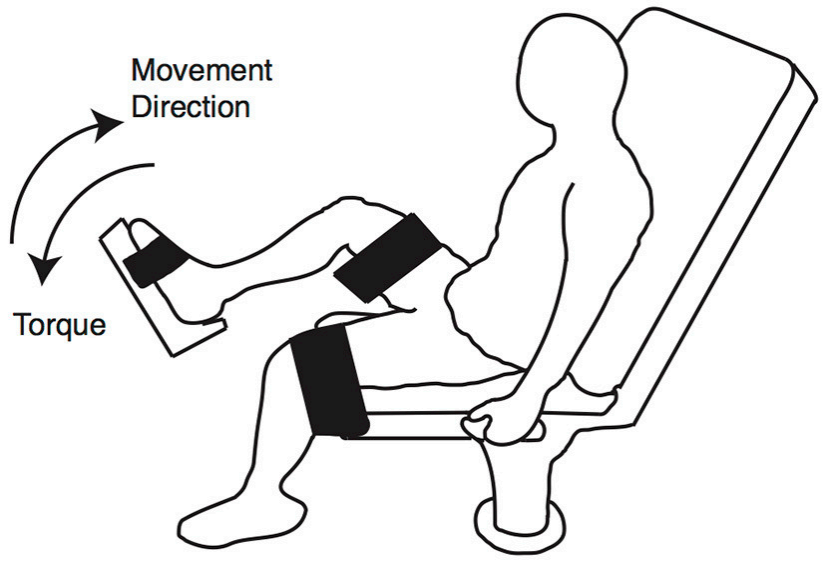
**MVIC and eccentric contraction positioning**. Subjects were seated on a dynamometer with the knee and hip joints at approximately 90°. Both legs were secured using straps.

Plantarflexion eccentric contractions and maximal voluntary isometric contractions (MVIC) were performed using a Humac Norm Dynamometer (CSMi, Stoughton, MA). Subjects were seated with the hip and knee joint of the tested leg at 90° and against the incline of the seat set to a comfortable position (Figure 3). Both of their thighs were secured to the dynamometer with straps during the course of testing.

MVICs were measured at the neutral position of the ankle. Subjects were allowed five practice trials and then executed three 5-s MVIC trials. During MVIC trials, they were instructed to ramp up torque as quickly as possible and hold the torque level for about 3 s while verbally encouraged. The highest torque value from the three trials was used to assess fatigue level as described below.

#### 2.3.5. Fatigue assessment

The percentage change in MVIC torque values between pre- and post-exposure was calculated to assess the presence of fatigue after eccentric contractions. Also, subjective assessments of fatigue (Borg scale) were obtained after eccentric contractions and walking. The Borg scale was scored between 0 and 10 (Grant et al., 1999).

### 2.4. Summary of testing protocol

**Figure 4:**
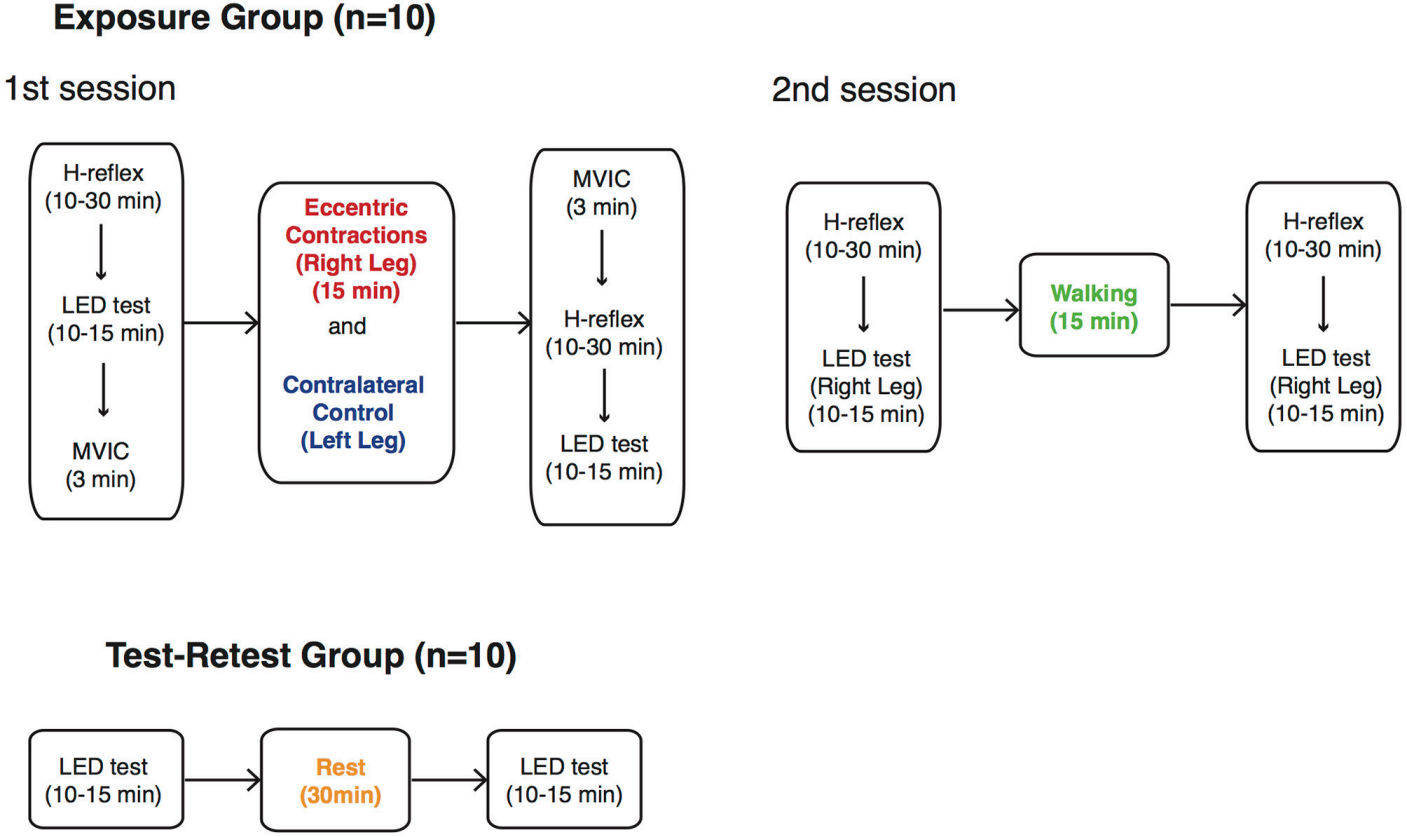
**Summary of testing protocol**. Ten subjects from Exposure group were exposed to two different interventions, eccentric contractions (ECC) and walking (WALK), in two separate sessions. During the first session, the contralateral leg remained at rest (CONTRA). Another set of 10 subjects (Test-Retest group) participated in an experiment where they performed the LED test bilaterally twice with 30-min rest in between. The order of measurements is shown with the duration required to complete each outcome measure.

The first 10 subjects (Exposure group) attended two separate sessions in which they were exposed to two different exercise modalities—eccentric contractions with the right leg (ECC) and walking (WALK)—after an initial baseline assessment of LED test performance (Figure 4). Subjects performed eccentric contractions with their right leg while their contralateral leg remained at rest (CONTRA). The eccentric contraction block began with a set of 10 familiarization trials followed by 500 eccentric contractions at 15% MVIC torque. This particular protocol was chosen to simulate the eccentric phase of walking (Winter, 1984) and the corresponding level of exertion, while minimizing possible fatigue effects (Corbeil et al., 2003; Gribble and Hertel, 2004; Wilkins et al., 2004; Springer and Pincivero, 2009). Completion of 500 repetitions required approximately 15 min. They were instructed to maintain the constant speed of eccentric movement provided by the dynamometer and to relax during period when the dynamometer brought the ankle back to the starting, plantarflexed position. To interrogate the specificity with which eccentric contractions may have mediated any possible behavioral changes, LED test performance was also obtained during the second session. This session consisted of walking on a level treadmill at individuals' preferred pace for the same duration as the individual spent to complete the eccentric loading protocol (approximately 15 min). For the WALK condition, the right leg was always the tested extremity. We chose not to randomize the order of exercise in order to match the activity level of walking to eccentric contractions for each subject.

As shown in Figure 4, H-reflexes were measured before and after the ECC and WALK phases to determine the degree to which eccentric contractions influenced the spinal processing of muscle afferent information. Only the right leg was tested for H-reflex measurements before and after walking because we expected that the symmetric, bilateral nature of the task would lead to similar effects on the H-reflex for both legs. We also measured MVICs of the plantarflexors to determine if there was evidence of fatigue after the eccentric contraction protocol. Subjects always completed all the measurements with their exposed leg first.

Based on observations from the first 10 subjects, another set of 10 subjects (Test-Retest group) was recruited to further investigate the neurophysiological mechanisms underlying changes in LED test performance. These subjects also had no prior experience with the LED test. In a single session, they performed the LED test bilaterally, twice with 30-min rest in between. LED test performance from this set of control subjects would allow us to once again confirm that there is no short-term learning effect as already reported in Lyle et al. (2013), or left-right difference in performance between legs as already reported in Lawrence et al. (2014).

### 2.5. Statistical analysis

To investigate effects of exposure to eccentric contractions and walking on LED_force_, LED_SD_ and H-reflex, we conducted two-way repeated measures ANOVA with time (pre- vs. post-exposure) and condition (ECC, CONTRA, and WALK) as factors. Recall that only the right leg was exposed to eccentric contractions. Likewise, another two-way repeated measures ANOVA tested for the effect of time (pre- vs. post-exposure) and leg (right vs. left) was used to test for effects of the 30-min rest period in the Test-Retest group. If significant main effects or interactions were found, paired *t*-tests were used for *post-hoc* analysis. Bonferroni corrections were applied to adjust significance level to take into account multiple comparisons. All the comparisons between conditions or between pre- and post-exposure are reported in mean ± SD in the text.

For other outcome measures from frequency domain analysis of LED_force_ and those related to EMG activity, Wilcoxon signed-rank test was used to test significant effects of exposure to eccentric contraction and walking by comparing between pre- and post-exposure values. We chose non-parametric test here because we observed non-normal distributions in the pre-post change scores in some of the outcome measures. Change scores between pre- and post-exposure are reported in median (25th to 75th quartile).

Significance level for all the statistical test performed in this study was set at *p* = 0.05. The statistical analysis described above was performed using MATLAB and statistical packages in R (v 3.1.1, R Development Core Team, 2010).

## 3. Results

### 3.1. Lower extremity dexterity

**Figure 5:**
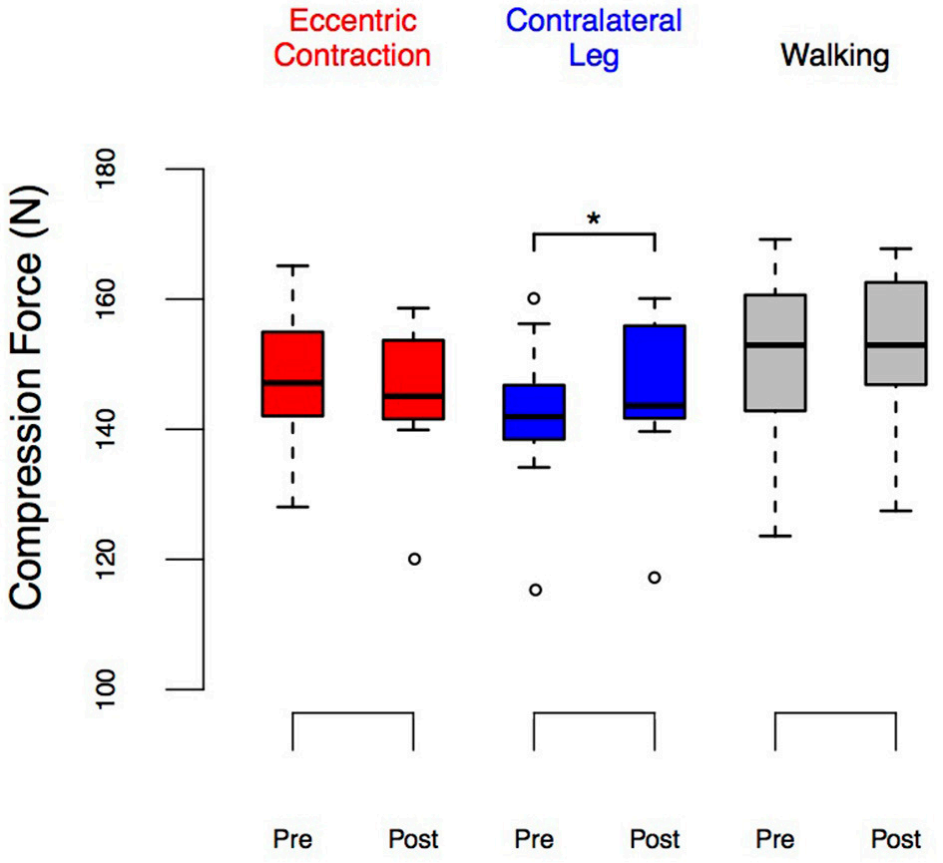
**LED_force_ before and after eccentric contractions or walking in the Exposure group**. Group performance is shown as box plots. The upper and lower ends of the box represent the upper and lower quartiles. The horizontal line inside the box represents the median. The whiskers show the range of adjacent values, representing the range of values not declared outliers. We observed a significant increase in LED_force_ in the contralateral leg (*p* = 0.037) and no changes in the exposed leg nor after walking. ^*^Denotes significant change (*p* < 0.05).

**Figure 6:**
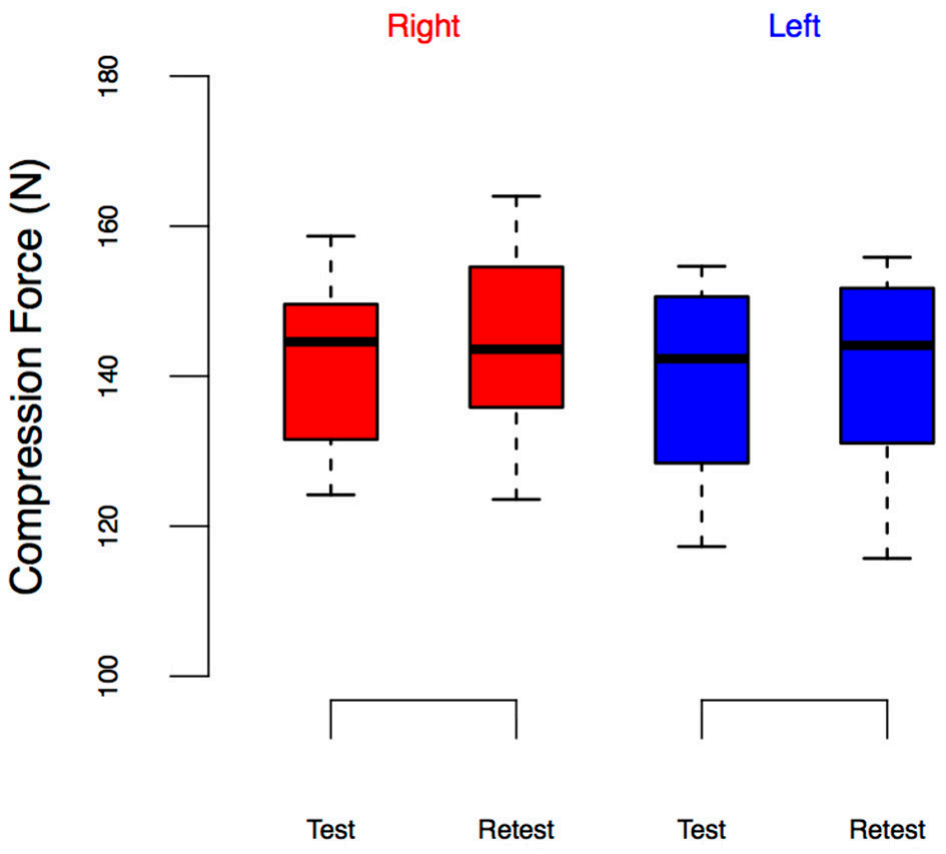
**LED_force_ from the Test-Retest Control group**. Group performance is shown as box plots. We did not observe leg-specific changes in LED_force_ at retest.

**Figure 7:**
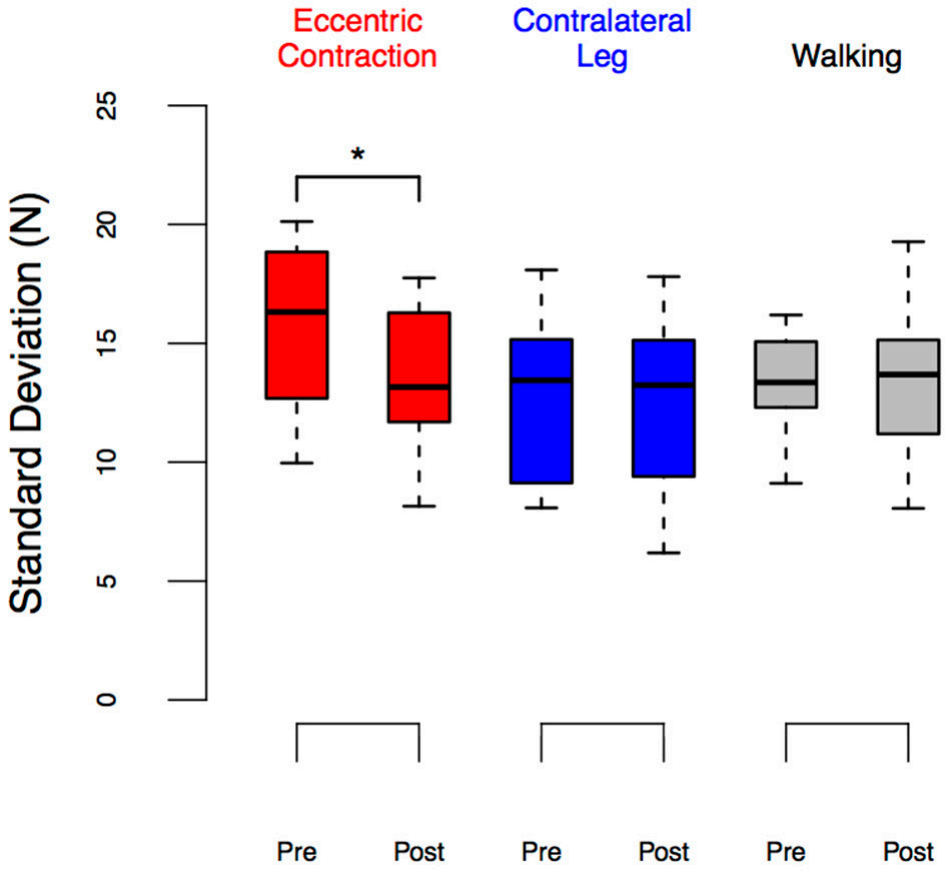
**LED_SD_ before and after eccentric contractions or walking in the Exposure group**. Group performance is shown as box plots. We observed a significant reduction in LED_SD_ in the leg exposed to eccentric contractions (*p* = 0.02) but no changes between pre- and post-exposure in other conditions. ^*^Denotes significant change (*p* < 0.05).

**Figure 8:**
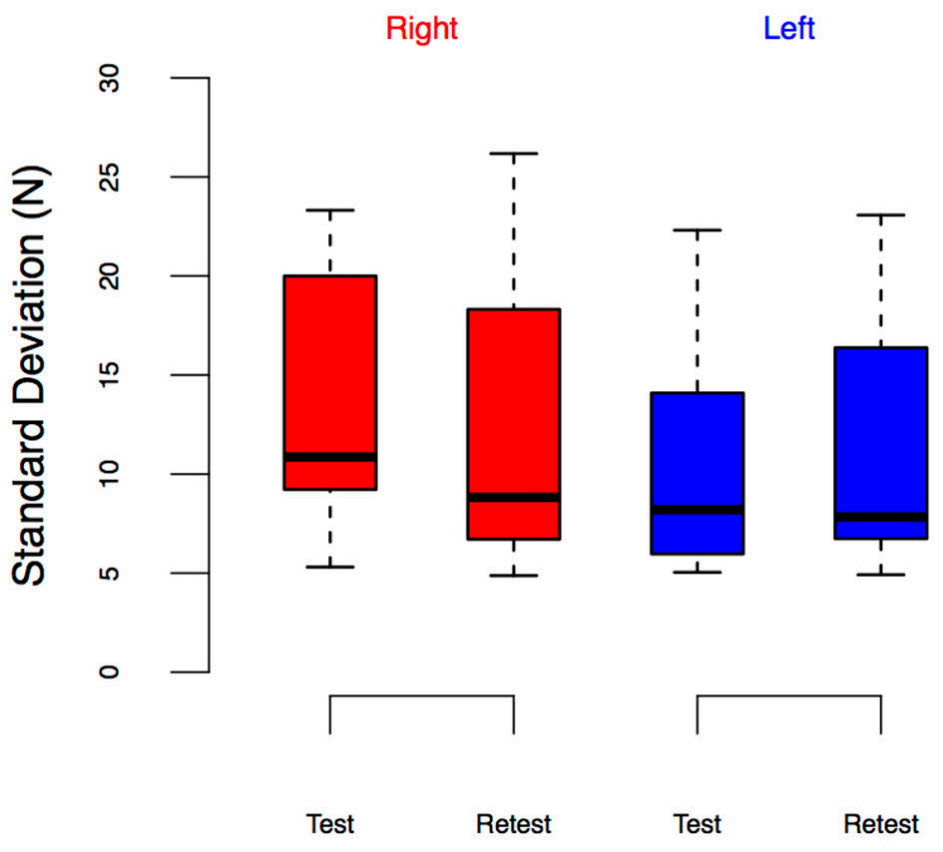
**LED_SD_ from the Test-Retest Control group**. Group performance is shown as box plots. We did not observe leg-specific change in LED_SD_ at retest.

**Figure 9:**
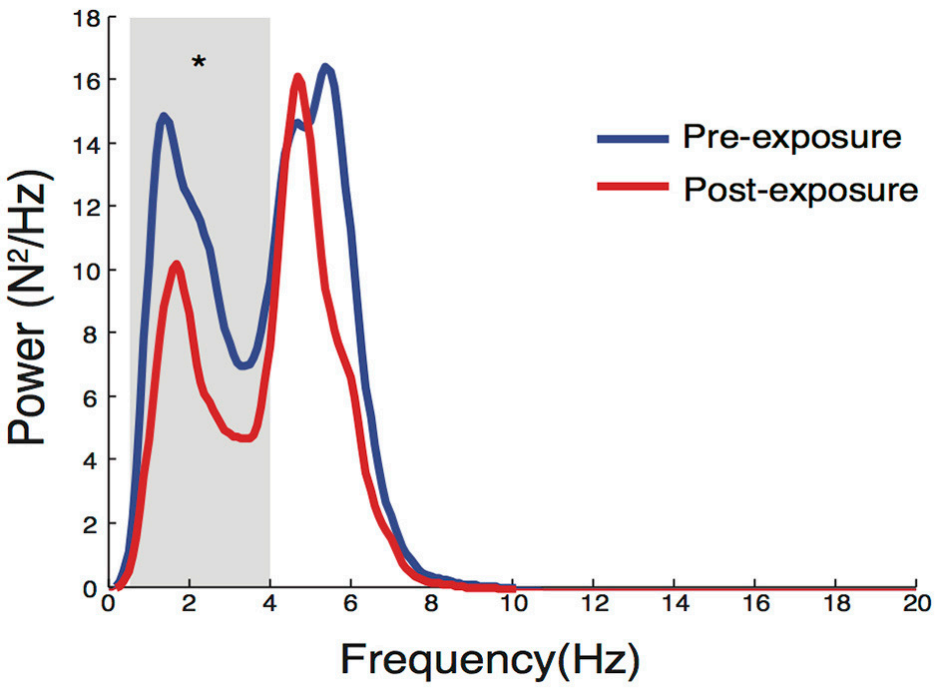
**Changes in power spectral properties in LED compression force after eccentric contractions**. There was a significant reduction in LED_0.5−4Hz_ (*p* = 0.027), while no significant change was observed in LED_4−8Hz_. ^*^Denotes significant change (*p* < 0.05).

Detailed results from all statistical analyses are provided in Table 1 and Figures 5–9.

**Table 1:** **Summary of repeated measures ANOVA results from the exposure group**.

**Variable**	**Main effect**		**Interaction**
	**Time Pre/Post**	**Condition ECC/CONTRA/WALK**	
LED_force_	*F*_(1, 9)_ = 0.66	*F*_(2, 8)_ = 20.33	*F*_(2, 18)_ = 4.95
	*p* = 0.44	^*^p < 0.01	^*^p = 0.019
LED_SD_	*F*_(1, 9)_ = 2.12	*F*_(2, 8)_ = 3.17	*F*_(2, 18)_ = 3.70
	*p* = 0.18	*p* = 0.097	^*^p = 0.045

* *Denotes significant effects (p < 0.05)*.

A repeated measures ANOVA test found no significant effect of time (i.e., pre/post, Table 1) on the LED_force_ results. However, it found a significant effect of condition (i.e., ECC, CONTRA, and WALK). [*F*_(2, 8)_ = 20.33, *p* < 0.01] on LED_force_, Figure 5, as well as an interaction effect [*F*_(2, 18)_ = 4.95, *p* = 0.019, Table 1]. This justified *post-hoc* pairwise comparisons to determine whether some conditions showed pre/post effects. Figure 5 shows that CONTRA was the only condition for which we saw pre/post effects (mean change score of 2.98 ± 3.82 N, *p* = 0.037, Figure 5). This suggests that the observed change in LED_force_ in the CONTRA limb was due to exposure to eccentric contractions.

Note that there seem to be visual differences across baseline LED_force_ values of the right leg between ECC and WALK conditions in Figure 5, but the *post-hoc* tests did not reveal a significant difference. To further explore the potential confound of learning/exposure effects, we tested a separate set of 10 subjects in which we performed a Test-Retest comparison in both legs, Figure 6. The repeated measures ANOVA for the Test-Retest group confirmed that the observed change in LED_force_ in the CONTRA limb were due to exposure to eccentric contractions, as we found no significant main effect of time [*F*_(1, 9)_ = 4.38, *p* = 0.07], leg [*F*_(1, 9)_ = 4.55, *p* = 0.06], or interaction [*F*_(1, 9)_ = 0.01, *p* = 0.91]. These results are consistent with prior work with many more subjects showing no difference between legs or exposure (Lawrence et al., 2014).

The results from the LED_SD_ values expand on these findings. Table 1 and Figure 7 show the results of that repeated measures ANOVA. It revealed that there were no significant main effects of time [*F*_(1, 9)_ = 2.12, *p* = 0.18] or condition [*F*_(2, 8)_ = 3.17, *p* < 0.01]. However, there was a significant interaction between time and condition [*F*_(2, 18)_ = 3.70, *p* = 0.045], which revealed a pre/post effect in the ECC condition only. Specifically, there was a significant decrease in LED_SD_ in the ECC limb after eccentric contractions (mean change score: −2.69 ± 2.27 N, *p* = 0.01, Figure 7), but no pre/post changes in LED_SD_ for the CONTRA or WALK limbs. As in LED_force_, results of the repeated measures ANOVA for the Test-Retest group confirmed that the observed change in LED_SD_ in the ECC limb were due to exposure to eccentric contractions, as we found no significant main effect of time [*F*_(1, 9)_ = 1.89, *p* = 0.20], leg [*F*_(1, 9)_ = 5.03, *p* = 0.05], or interaction [*F*_(1, 9)_ = 0.38, *p* = 0.55].

Further analysis of LED_force_ in the frequency domain revealed that the reduction in LED_SD_ in the ECC limb after exposure to eccentric contractions occurred only in the 0.5–4 Hz band [median change scores with interquartile ranges of −3.2 (−5.7 to −1.4) N^2^/Hz, *p* = 0.027, Figure 9]. Eccentric contractions did not, however, alter power of LED_force_ in the 4–8 Hz band [0.2 (−4.4 to 1.8) N^2^/Hz, *p* = 0.770]. This reduction strongly suggests that subjects altered the long-latency/voluntary feedback control of dynamic foot-ground interactions (Squeri et al., 2010).

### 3.2. Muscle activity

Table 2 shows the results of the change scores for EMG of the soleus muscle. Recall that we did not use repeated measures ANOVA because EMG signals across legs (i.e., ECC and WALK in the right leg, CONTRA in the left leg) cannot be compared in the absence of an objective normalization. We found change scores significantly different from zero (i.e., interquartile ranges did not include the zero) only for the physiological tremor band (EMG_4−8Hz_) in the ECC condition. In the TA muscle, EMG signals showed significant reductions in its power (EMG_RMS_) (Table 3). No EMG values showed significant changes in CONTRA or WALK conditions (Tables 2, 3).

**Table 2:** **Summary of EMG variables for soleus**.

**Condition**		**EMG_RMS_ (mV)**	**EMG_0.5−4Hz_ ×10^-3^ (AU)**	**EMG_4−8Hz_ ×10^-3^ (AU)**
*ECC*	Pre	20.7 (16.3–33.6)	10.3 (6.1–12.5)	7.2 (6.1–9.2)^*^
	Post	16.9 (13.5–34.2)	7.7 (7.0–8.7)	5.8 (5.0–7.1)^*^
*CONTRA*	Pre	15.9 (9.8–24.5)	7.5 (4.3–10.4)	4.8 (4.0– 7.1)
	Post	17.8 (10.9–25.6)	7.5 (5.0–10.1)	5.6 (4.1–8.3)
*WALK*	Pre	20.4 (13.5–30.4)	9.2 (7.1–9.6)^*^	6.6 (5.9– 6.8)
	Post	21.8 (13.8–25.8)	7.7 (6.2–13.4)^*^	5.8 (4.8–7.2)

*Raw values are shown as median (interquartile range). Note that only EMG_4−8Hz_ shows significant effects (Wilcoxon signed-rank test)*.

* *Denotes significant effects (p < 0.05)*.

**Table 3:** **Summary of EMG variables for TA**.

**Condition**		**EMG_RMS_ (mV)**	**EMG_0.5−4Hz_ ×10^-3^ (AU)**	**EMG_4−8Hz_ ×10^-3^ (AU)**
*ECC*	Pre	93.6 (78.9–194.6)^*^	8.9 (6.1–13.9)	4.9 (3.3–6.6)
	Post	81.2 (61.9–101.0)^*^	7.8 (6.2–8.9)	4.4 (2.7–6.3)
*CONTRA*	Pre	83.6 (45.8–101.6)	4.8 (3.6–5.7)	4.4 (3.5– 5.0)
	Post	73.1 (41.0–95.1)	5.1 (3.6–6.0)	4.8 (3.0–5.9)
*WALK*	Pre	95.0 (82.5–122.1)	5.5 (5.2–6.7)^*^	4.9 (4.1– 5.6)
	Post	84.2 (71.5–115.5)	4.7 (3.8–5.9)^*^	4.1 (3.6–6.7)

*Raw values are shown as median (interquartile range). Note that EMG_RMS_ shows significant effects (Wilcoxon signed-rank test) in the ECC limb and EMG_0.5−4Hz_ in the WALK condition*.

* *Denotes significant effects (p < 0.05)*.

In addition, the level of cocontraction between the soleus and TA during the LED test did not show pre/post changes in any condition [median change scores with interquartile ranges of 0.035 (−0.013 to 0.086), 0.017 (−0.026 to 0.071), and 0.008 (−0.013 to 0.068) for ECC, CONTRA, and WALK, respectively].

### 3.3. Hoffmann reflex

We did not observe significant effects of ECC or WALK conditions on resting H-reflex response (H/M_max_) of the soleus. Repeated measures ANOVA showed no significant main effect of time [*F*_(1, 9)_ = 0.04, *p* = 0.84], condition [*F*_(2, 8)_ = 1.00, *p* = 0.41], or interaction [*F*_(2, 18)_ = 3.60, *p* = 0.08]. This result suggests that neither ECC nor WALK affected resting spinal excitability.

### 3.4. Fatigue

We did not observe any signs of fatigue after eccentric contractions as per MVIC (mean percentage increase of 18.8 ± 23.6%) and Borg scale ratings of perceived exertion (mean value of 2.0 ± 0.7 out of 10).

## 4. Discussion

The primary purpose of this study was to investigate effects of repeated eccentric contractions on the ability to regulate dynamic foot-ground interactions as measured by an adaptation of the Strength-Dexterity test to evaluate leg dexterity (Valero-Cuevas et al., 2003; Lyle et al., 2013; Lawrence et al., 2014). Performance in this LED test was quantified by the maximal sustained compression force (LED_force_) of a slender spring, and its standard deviation (LED_SD_) during the hold phase. To our knowledge, this is the first study to report changes in the ability to regulate dynamic foot-ground interactions due to exposure to an eccentric exercise intervention. Specifically, low-intensity, repetitive eccentric contractions resulted in a decrease in LED_SD_, a measure of force variability during the control of dynamic foot-ground interactions at low force magnitudes c. 20% MVIC. Interestingly, we also observed an increase in LED_force_ in the *contralateral, non-exposed leg* after this unilateral eccentric exercise. We also found that level walking for the same duration and at similar intensity as eccentric contractions did not induce a change in LED test performance. These results suggest that unique physiological processes specific to eccentric contractions led to alterations in the sensorimotor processing required to regulate dynamic foot-ground interactions. It is importance to note that potential effects of MVIC trials were one of the confounding factors in our study. However, we think that a relatively small number of repetitions for MVIC (six repetitions in total before and after eccentric contractions) compared to 500 repetitions of eccentric contractions would have minor effects.

It has been shown previously that the dynamics of LED performance can be used to identify differences in leg dexterity across different populations (Lawrence et al., 2014). Here, we quantified overall force variability by LED_SD_ and analyzed LED_force_ in the frequency domain. The significant reduction in LED_SD_ —and its independence from the change in LED_force_—in the leg exposed to eccentric contractions suggest eccentric contractions altered the way in which dynamic foot-ground interactions are controlled. Interestingly, this reduction in force variability occurred primarily in the 0.5–4 Hz band, which is thought to reflect long-latency/voluntary control (Squeri et al., 2010). This may indicate that subjects reduced the amount of long-latency/voluntary intervention to regulate the dynamics of the unstable foot-ground interactions. This can be considered beneficial as it likely suggests a reduction of supraspinal or cortical involvement in the control of dynamic and unstable foot/ground interactions.

Moreover, contrary to previous reports in different paradigms (Finley et al., 2012), but in agreement with other reports with this paradigm (Lyle et al., 2013; Lawrence et al., 2014), we did not observe an increase in cocontraction as a stabilizing strategy, which suggests that a reactive neural control strategy is being used, in contrast to the exclusive use of a general, feed-forward impedance control strategy. Passive impedance or viscoelasticity of the limb clearly contribute to short-latency stabilization as we have mentioned before (Lawrence et al., 2014), but the results here confirm the involvement of active neural control (Ko et al., 2015). Together, these results suggest that changes in LED performance in the leg exposed to eccentric contractions may have been mediated through changes in the relative contributions of short-latency, spinal feedback circuits, and long-latency/voluntary control.

A change in the gain of the spinal stretch reflex pathway due to eccentric contractions is one plausible factor that could result in differential effects of eccentric contractions and walking on feedback control of lower extremity dexterity (Lawrence et al., 2014). This study further clarifies those mechanisms. Specifically, sensory information originating from muscle spindles likely plays an important role in the face of continuous perturbations, where movement corrections according to state of the body are critical. The gain of the spinal stretch reflex pathway can be quantified indirectly by the power contained in oscillations in either force or rectified EMG signals in the 4–8 Hz band (i.e., physiological tremor) (Iaizzo and Pozos, 1982; Pozos et al., 1982). We found a reduction in the power of soleus EMG within the physiological tremor band, but did not observe changes in its spinal excitability as measured by the amplitude of the H-reflex. This suggests that changes in reflex gain were mediated by changes at the level of the muscle spindle itself (and not the strength of its homologous monosynaptic projections onto its α-motoneuron pools). Consistent with this possibility, previous findings showed that repeated stretching can lead to a prolonged (>15 min) reduction in stretch reflex amplitude, but not in H-reflex amplitude (Avela et al., 1999, 2004). This presumably results from an increase in the compliance of series elastic elements (Kubo et al., 2001a,b), thereby decreasing the mechanical transduction of stretch at the level of the muscle spindles. This mechanism may also explain the observations of the current study, particularly since we did not observe changes due to level-ground walking which is a concentric-biased movement for the plantarflexors (Winter, 1983; Neptune et al., 2001) and would therefore have produced less muscle stretch.

If changes in spindle function were, in fact, altered, how would this affect the control of dynamic foot-ground interactions? Given that the spinal stretch reflex pathway involves transmission delays which have the potential to induce instabilities, reducing feedback gains could be interpreted as an intelligent strategy to limit delay-induced instability, particularly in tasks that require rapid adjustments. Consistent with this idea, previous studies have shown that short- and medium-latency reflex responses of the ankle extensors are attenuated during control of unstable loads (Finley et al., 2012, 2013). Although those studies suggested that increased levels of cocontraction were a potential mechanism responsible for reductions in the gain of spinal stretch reflex pathway, it seems reasonable to speculate that the attenuation of spinal stretch reflex gain due to increased compliance of muscle-tendon complex would have helped to reduce the potentially detrimental effects of delayed feedback on control of dynamic foot-ground interactions.

It might also be possible that eccentric contractions, but not walking, altered supraspinal feedback control of dynamic foot-ground interactions, as indicated by the reduction in force variability in the 0.5–4 Hz. Previously, voluntary contractions with augmented sensory inputs through vibration, in many studies, have been found to improve the efficacy of transmission between sensory input and motor output (Siggelkow et al., 1999; Rosenkranz and Rothwell, 2004, 2006; Marconi et al., 2008). The higher degree of muscle lengthening during eccentric contractions gives rise to increased Ia afferent activation compared to other contraction types (Burke et al., 1978). Therefore, improved transduction of sensory input to motor output due to eccentric contractions may have contributed to the improved ability to regulate dynamic foot-ground interactions. The absence of these effects following walking may be due to the bias toward concentric contractions of the platarflexor muscles (Winter, 1983; Neptune et al., 2001) and a suppression of cortical excitability compared to that observed during voluntary isometric contraction (Capaday et al., 1999).

Most interestingly, we observed an increase in LED_force_ (i.e., improved dexterity) in the resting contralateral leg, opposite to the one exposed to eccentric contractions. We have previously shown no left/right differences in leg dexterity (Lawrence et al., 2014). But it is possible that the improvement in LED_force_ in the contralateral leg was attributable to learning effects, use of the left, non-dominant leg as the contralateral leg, or recruitment of subjects who were novice to the LED test. To address these possibilities, we recruited a control set of 10 subjects who had no prior experience with the LED test and had them perform the test bilaterally twice with 30-min rest in between (Test-Retest control group). The results showed that exposure alone did not lead to improvements in LED_force_ in either leg, consistent with previous findings (Lyle et al., 2013; Lawrence et al., 2014). This suggests that unilateral, repetitive eccentric contractions of one leg might have distinct neurophysiological effects on the *contralateral leg*.

A possible explanation for the improved performance in the contralateral leg is central or peripheral neural adaptations due to repeated eccentric contractions. For example, eccentric, but not concentric, contractions can lead to an acute improvement in strength of the contralateral limb (Grabiner and Owings, 1999). Additionally, studies on chronic (≈ 6-week) neuromuscular adaptation due to strength training have shown that eccentric training leads to greater improvement in strength, EMG amplitude and voluntary activation in the contralateral limb compared to concentric training (Hortobágyi et al., 1997). It seems unlikely that reported neuromuscular adaptations specific to eccentric contraction had their origin at the spinal level, as evidenced by the absence of systematic differences in changes in H-reflex amplitudes between eccentric and concentric training (Hortobágyi et al., 2003; Howatson et al., 2011). The results from our study are consistent with these observations, showing no significant changes in the power of the soleus EMG in the physiological tremor band during the LED test or in resting H-reflex response in the contralateral leg. Therefore, these results point to changes at the supraspinal level as the mediating factor leading to performance improvements in the contralateral leg.

In line with this idea, it has been postulated that the cortical control strategies required for eccentric contractions contribute to the supraspinal adaptations responsible for improvements in performance after eccentric exercise (Howatson et al., 2011). Howatson and colleagues showed greater increases in ipsilateral motor cortex excitability and greater reductions in intracortical and interhemishperic inhibition to the ipsilateral motor cortex during eccentric contractions compared to concentric contractions. Furthermore, eccentric contractions involve more extensive neural networks than concentric contractions, including frontal and parietal areas associated with movement planning, executive function, and processing of sensory feedback (Kristeva et al., 1991; Fang et al., 2001). Indeed, increased activity of these networks has been observed during dexterous manipulation of dynamic interactions with unstable objects with the thumb and index fingers (Mosier et al., 2011). These results suggest that changes in the communication between multiple brain areas might have contributed to the improved ability to regulate dynamic foot-ground interactions in the contralateral leg.

Lastly, the observed bilateral improvements in leg dexterity after low-intensity, repeated eccentric contractions may also have athletic and clinical implications. Our previous studies have shown that leg dexterity is associated with various functional outcome measures such as landing biomechanics, agility, and skiing ability (Lyle et al., 2014, 2015; Krenn et al., 2015; Lawrence et al., 2015a). This study is, to our knowledge, the first to report improvements in leg dexterity due to an acute bout of eccentric exercise, which suggests that eccentric exercise could potentially be used as an intervention to improve leg dexterity. For example, warm-up protocols that involve repeated eccentric contractions might help prevent non-contact ACL injuries (Lyle et al., 2014). Also, training or rehabilitation regimens that incorporate eccentric contractions could potentially improve leg dexterity in individuals with a previous history of ankle sprains and thereby reduce the likelihood of recurring sprains (Kobayashi and Gamada, 2014). Furthermore, contralateral facilitation of leg dexterity due to eccentric exercise might facilitate rehabilitation in individuals with hemiparesis. In those individuals, both feedback and anticipatory control of the more affected limb are disrupted, resulting in impaired postural control and mobility limitations (Badke and Duncan, 1983; Di Fabio, 1987). Although this impaired postural control can be improved through balance training, disrupted sensory, and motor function of the more affected side often limits active interventions to promote recovery of such function (de Haart et al., 2004). Our results, together with previous findings, suggest that unilateral eccentric exercise of the less affected limb might facilitate earlier and more rigorous intervention, accelerating recovery of leg dexterity and mobility.

## Author contributions

AN designed study, performed data collection, analyzed and interpreted data, and wrote manuscript. FVC designed study, analyzed and interpreted data, and wrote manuscript. JMF designed study, analyzed and interpreted data, and wrote manuscript.

## Funding

This study was supported by the National Institute of Arthritis and Musculoskeletal and Skin Diseases of the National Institute of Health (NIH) under award numbers R01AR050520 and R01AR052345 grants to FVC and by the Eunice Kennedy Shriver National Institute of Child Health & Human Development of NIH under award number K12HD073945 to JMF.

### Conflict of interest statement

FVC holds US Patent No. 6,537,075 on some of the technology used in this study that is commercialized by Neuromuscular Dynamics, LLC. AN, JMF have no financial or personal relationships with other people or organizations that could inappropriately influence this work.
